# The Influence of Ni Addition in the Mechanism of CO_2_ Electroreduction on Cu Crystals—Mechanistic Insight from DFT Simulations

**DOI:** 10.3390/ma16145138

**Published:** 2023-07-21

**Authors:** Elżbieta Dziadyk-Stopyra, Ionut Tranca, Daniel Smykowski, Bartłomiej M. Szyja

**Affiliations:** 1 Institute of Advanced Materials, Faculty of Chemistry, Wrocław University of Science and Technology, Gdańska 7/9, 50-344 Wrocław, Poland; elzbieta.dziadyk@pwr.edu.pl; 2 Algemene Chemie, Vrije Universiteit Brussel, Pleinlaan 2, B-1050 Brussels, Belgium; ionut.tranca@vub.be; 3 Department of Energy Conversion Engineering, Faculty of Mechanical and Power Engineering, Wrocław University of Science and Technology, Wybrzeże Wyspiańskiego 27, 50-370 Wrocław, Poland; daniel.smykowski@pwr.edu.pl

**Keywords:** CO_2_ reduction, electrocatalysis, DFT, CuNi, mechanisms

## Abstract

We present a DFT analysis of the role of the Cu-Ni synergistic effect for the CO_2_ reduction to C_2_H_4_, in comparison to the pure Cu catalyst. The analysis is focused on the thermodynamic stability of reactive intermediates along the proposed pathway of C_2_ species formation. We have observed that the potential needed for the reaction decreases with the addition of Ni in the investigated model. In addition, we have observed the differences in the preferred pathway based on the significant differences in stability of the reactive intermediates depending on th Cu:Ni ratio. The results suggest that despite the fact the Cu surface is always exposed, and it is the only one that is able to directly interact with the intermediates, the presence of the Ni in the underlying sections of the crystal is significant enough to change the mechanism of the reaction.

## 1. Introduction

Numerous solutions have been proposed to address the problem of constantly increasing CO_2_ content in the atmosphere [1,2,3], among which catalytic conversion to value-added chemicals seems to be the most promising [4]. Chemical or electrochemical conversion of CO_2_ into fuels, coupled with the use of renewable energy, resolves two essential issues—utilization of CO_2_ with subsequent renewable energy storage in green, synthetic fuels [5,6]. Such conversion, however, is difficult due to the high thermodynamic stability of a CO_2_ molecule, which requires energy input for a conversion [1,2,4]. From an environmental point of view, the only sustainable solution is to use green energy from renewable sources—for instance, in a process directly using sunlight [7]. Another—more versatile—solution is the electrocatalytic CO_2_ reduction, in which not only solar cells, but other renewable energy resources—such as wind- or hydropower—can be utilized to supply direct electrical energy for the CO_2_ conversion process [8].

As far as electrocatalytic conversion CO_2_ is concerned, copper electrodes seem to have unique properties compared to other metals with respect to CO_2_ reduction [9]. Cu is the only pure metal capable of reducing CO_2_ to C_2_^+^ hydrocarbons, which requires the transfer of more than two electrons. The other metals can be divided into three groups—Pb, Hg, Tl, In, Sn, Cd and Bi produce primarily formates; Au, Ag, Zn, Pd, and Ga—carbon monoxide; and Ni, Fe, Pt, and Ti almost exclusively reduce protons to H_2_, and thus are inert with respect to the CO_2_ reduction [10]. In a similar way to any other catalytic reaction, the selectivity of the process on different metal electrodes is a result of the differences in the interaction energies with the reacting species [11].

The amount of work devoted to the development of new electrocatalytic systems is extensive, and for a more comprehensive review, the reader is referred to the other works [10,12,13,14]. Here, to put our work in the right context, we will focus on the nanostructurization of the Cu-based catalysts.

Bu et al. [15] demonstrated the coupling of size and symmetry of several Cu clusters as well as temperature on CO_2_ reduction. The authors found that decreasing the size of a cluster is beneficial, reducing CO_2_, in icosahedron-shaped clusters, but disadvantageous in truncated octahedron clusters. They also concluded that smaller (111)-like surfaces, surfaces of Cu_13_ and Cu_55_, are preferred in hydrocarbon production at low potential (*U* = −0.43 V), while the synergistic effect between (100) and (111)-like ones on Cu_38_ and Cu_79_ is favorable to CO production at the potential of *U* = −0.59 V.

Dong et al. [16] performed a DFT analysis of Cu-based systems and found that the Cu_79_ nanoparticle (NP) exhibits a stronger binding to reaction intermediates than the Cu(111) and Cu(211) surfaces. Importantly, according to the authors, a trend of the onset potentials is correlated with adsorption energetics of COOH, CO, and CHO.

Unfortunately, the size and shape of the supported Cu clusters is difficult to control experimentally, and often depends on the support used. Xu et al. [17] have used operando X-ray absorption spectroscopy in order to identify a reversible transformation from atomically dispersed Cu atoms to Cu_n_ clusters (n = 3 and 4) in electrochemical conditions. Furthermore, they report high selectivity to ethanol with Faradaic Efficiency (FE) of 91% at −0.7 V.

Another option for improving Cu systems is to alloy it with another metal [18], which allows alteration of both the electronic and geometric structures of the catalytically active surface sites. The electronic structure of the catalyst directly influences the binding energies of the intermediates, which affects the reaction pathway. However, the local atomic arrangement at the active site can exhibit a favorable or unfavorable effect for certain intermediates and effectively alter the reaction pathway. Copper–gold nanoparticles have been investigated by Kim et al. [19]. The authors found that there is a synergy between the electronic and geometric structure of these nanoparticles, which allows us to increase the catalytic activity. Li et al. [20] investigated the Cu−Pd alloys and concluded that optimal results have been obtained with the Pd_7_Cu_3_ systems. The authors claim that the selectivity to CO can be obtained with 80% FE. Importantly, the synergistic effect of the electronic and geometric structures, which improves the catalytic properties, has also been confirmed in their work. However, it should also be mentioned that the synthesis of surfaces or NPs of Cu alloys is even more difficult to control experimentally. The size of NPs is not only subject to distribution [21,22], but also the ratio of one metal to another may vary [23].

As many as 16 different products of the electrocatalytic reduction of CO_2_ have been experimentally identified [9,24], which only emphasizes the complexity of the reaction mechanism. In addition to hydrocarbons, many partially oxidized species have been identified, such as aldehydes or ketones [24]. Interestingly, methanol is not a product in the electrocatalytic process, despite the fact that Cu is the key component of the conventional catalyst for methanol synthesis from CO_2_ [25].

The pathways of the electrocatalytic conversion of CO_2_ on pure Cu surfaces have been theoretically investigated by Peterson et al. [26]. The authors concluded that the key step in the formation of hydrocarbons from CO_2_ is the formation of CHO species. Moreover, they hypothesize that if adsorbed −CHO can be stabilized relative to adsorbed CO, the overpotential of the process can be significantly reduced.

Importantly, with respect to C_1_ products, compounds containing more C atoms in their structure are characterized by higher energy density [27]. Until now, only Cu materials have demonstrated the capability of C−C coupling to form C_2_ and C_3_ hydrocarbons and oxygenates, although with low selectivity [28]. It is generally accepted that the breaking of the scaling relationships in order to stabilize the particular intermediates is required to lower the overpotential and influence selectivity can be achieved by alloying Cu with other metals [27,29]. The exact role of the addition of the other metal depends on many factors: mostly the type of the additive, its content and the nanostructure being formed [27].

## 2. Aim and Scope

In this work, the electrocatalytic properties of the pure Cu surface and CuNi alloys are investigated for the electroreduction of CO_2_ to ethylene (C_2_H_4_). Currently there is little consensus on the stage of the C−C bond formation, especially in the case of bimetallic alloys [30]. Hence, we investigate the pathway in which the oxalate (C_2_O_4_^2-^) is initially formed during dimerization of two CO_2_ molecules, co-adsorbed on the catalyst surface. In addition, we also discuss the binding of two −CHO intermediates, as suggested in the literature [31].

Furthermore, our objective is to verify the synergistic effect of copper and nickel metals in the CO_2_ electroreduction. Such modifications were selected to consider the influence of Ni while maintaining the direct contact of the Cu with reactive species in order to determine the selectivity of the process. To the best of our knowledge, the effect of underlying Ni on the interactions between the reactants and Cu surface has not been investigated with respect to the investigated process.

## 3. Model and Computational Details

**Figure 1:**
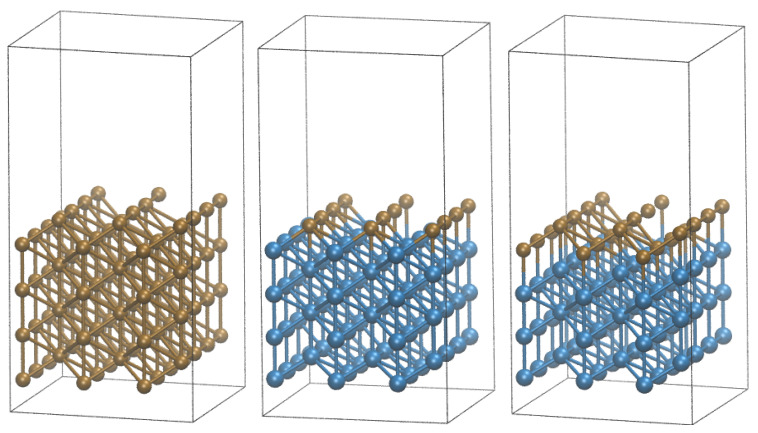
Optimized models used in simulation (**Left**) pure Cu surface, (**Middle**) 1 Cu monolayer (1 ML) on top of Ni, (**Right**) 2 Cu monolayers (2 ML) on top of Ni. The brown spheres represent the Cu atoms and blue—Ni atoms.

The initial model is shown in Figure 1 left. It consists of eight monolayers of Cu(110) surface. The periodic boundary conditions were imposed by means of a box of 10.6 × 9.9 × 20.7 Å lengths and angles of $\alpha =\beta =\gamma =90^{\circ }$. The vacuum slab of 12 Å thickness above the Cu surface was used to avoid any interactions between periodic images in the *c* direction.

To investigate the role of Ni, two additional models have been constructed, in which the initial model has been modified by replacing all but one and two layers (see Figure 1) of Cu atoms with Ni. Thus, these systems also consisted of a total of eight layers, with Cu layer(s) on top of each model.

All calculations have been carried out within the Density Functional Theory (DFT) framework, as implemented in the VASP (Vienna Ab initio Simulation) code ver. 5.4.4 [32,33]. The Perdew–Burke–Ernzerhof [34] functional was used to describe the exchange correlation energy, and the plane wave energy cutoff was set at 400 eV. The electron–ion interactions were described by the projector-augmented wave method [33,35]. The Brillouin zone was sampled by 2 × 2 × 1 *k*-point mesh generated using the Monkhorst–Pack method [36]. The dipole moment corrections have been used in order to avoid any artificial polarization.

**Figure 2:**
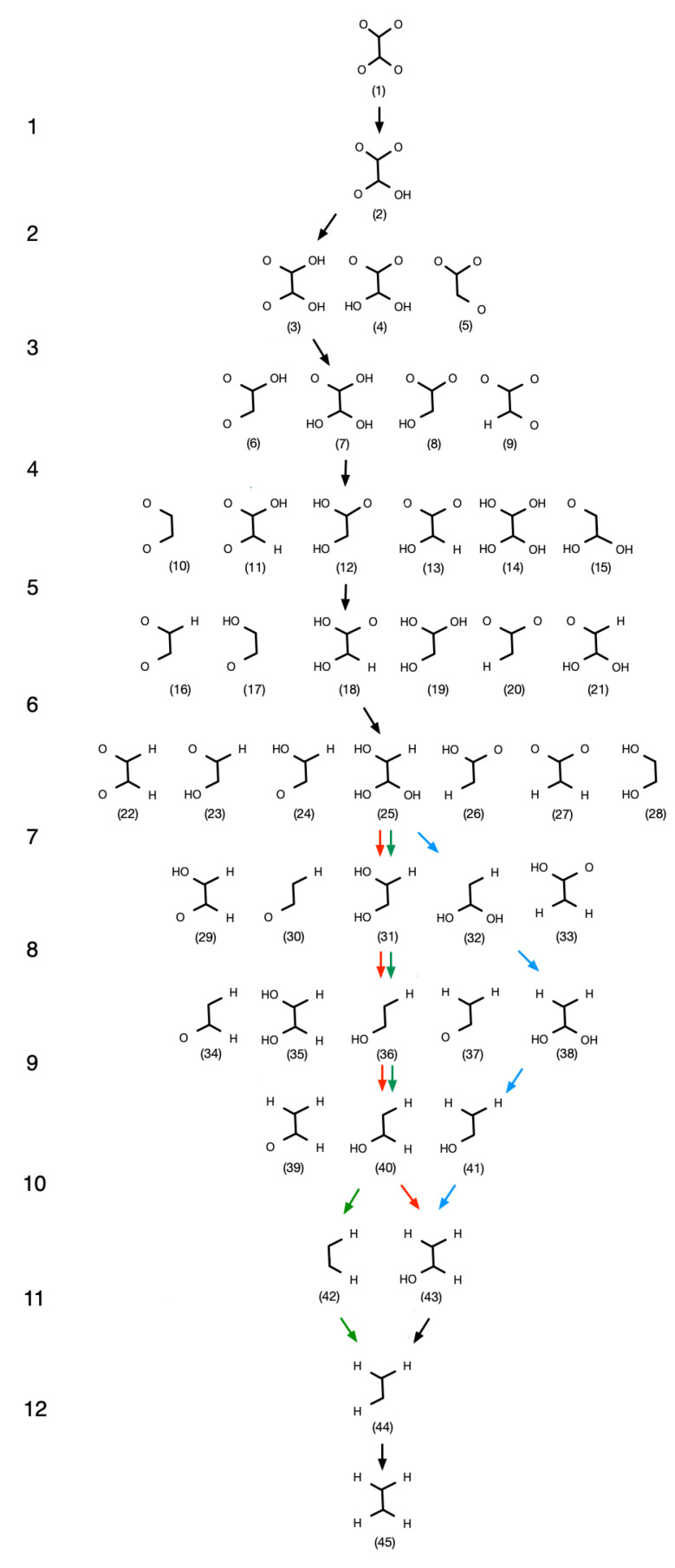
Complete transformation network for the electroreduction of C_2_O_4_ to C_2_H_4_ product with color-coded lowest energy pathways on Cu (red—pathway number I), 1 ML CuNi (green—pathway II) and 2 ML CuNi (blue—pathway III) catalysts.

For each of the possible intermediates shown in Figure 2, we have carried out geometry optimization in order to determine the relative stability with respect to the other intermediates. Structures were optimized to reach the forces on each atom smaller than 0.01 eV/Å. In order to account for vibrational entropy, the Zero Point Energy (ZPE) corrections have been calculated according to the finite displacement method, with the displacement of 0.0015 Å, as implemented in VTST [37]. Thus, the energies discussed in this work have the meaning of Helmholz Free Energies in 0 K.

The graphs represent the most essential steps of the catalytic cycle, while the phenomena that do not contribute to the calculation of the potential of the process—such as adsorption of the reactants or desorption of the product—have been omitted. These steps are not potential dependent, and as such they will not be influenced by any applied bias. Our analysis focuses on the thermodynamics of the intermediates conversion as well as the interactions with the cathode surface, which is responsible for the thermodynamic limitations of the entire process.

**Figure 3:**
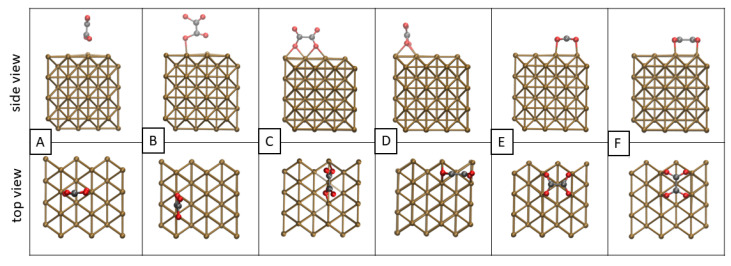
Side and top views of various adsorption modes on the example of C_2_O_4_ on the pure Cu surface. (**A**): frontal perpendicular across the rows; (**B**): frontal perpendicular along the rows; (**C**): lateral perpendicular along the rows; (**D**): lateral perpendicular across the rows; (**E**): parallel across the rows; (**F**): parallel along the rows.

For each of the intermediates investigated, six modes of adsorption with different orientations of the molecule were analyzed with respect to the direction *x* of the cell: vertical up (Figure 3A,B), vertical side (Figure 3C,D) and horizontal (Figure 3E,F).

The pathway diagram has been created based on selection of the most thermodynamically favorable structures for each intermediate stage. The investigated mechanism includes 45 structures which provide hundreds of possible pathways. To simplify the analysis and reduce computational demand, we have discarded pathways that involved less stable intermediates as unfavorable ones.

To account for solvent, single-point energy calculations (Supplementary Material Tables S1–S3) have been carried out on the optimized geometries of all intermediates, along with the continuous solvent model as implemented in VASPsol [38].

The electrocatalytic process has been investigated using a procedure proposed by the group of Nørskov [26], which relates the transfer of the proton/electron pair to the potential of the hydrogen electrode in the equilibrium in the standard conditions. The free energies for each electroreduction step were calculated using Formula (1):

(1) $$\Delta G=\Delta G_{product}-\Delta G_{reactant}-eU$$

in which ΔG is the free energy of formation of the particular intermediate, *e* is elementary charge and *U* is the potential. This equation assumes the linear dependence of the applied potential on the Gibbs free energies. In addition, the method assumes the equilibrium of the 2H^+^ + 2e^−^ ⇌ H_2_ reaction, which allows us to calculate the energy of a proton/electron pair as 12 of the hydrogen molecule in the gas phase.

The potential limiting step of the reaction has been calculated as the minimum potential required to make all the consecutive reaction steps preserve the descending energy trend.

Partial charges on atoms and bond orders have been calculated using the DDEC6 scheme [39,40,41].

## 4. Results

### 4.1. Pathways

**Figure 4:**
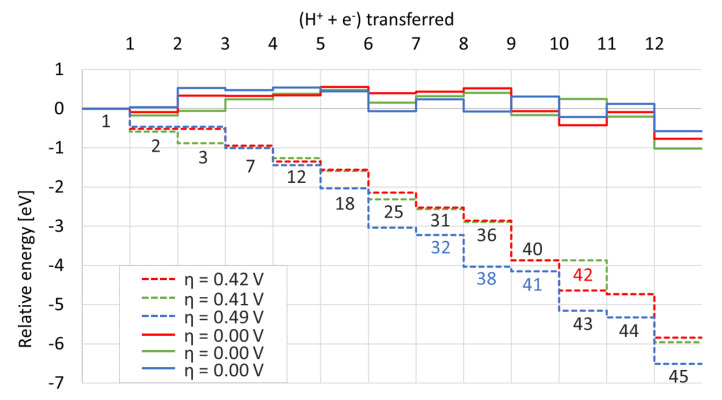
Comparison of free energy profiles for the pathway to C_2_ species on Cu (red, pathway I), Cu_1_Ni_7_ (green, pathway II) and Cu_2_Ni_6_ (blue, pathway III). The color of the respective data series is consistent with the colors of the particular dashed pathways in Figure 2.

The pathways of the CO_2_ hydrogenation to ethylene are shown in Figure 2. Each row of reactants represents a consecutive step of proton/electron pair transfer; in particular, one of the C or O atoms forms a bond with a proton and an electron is transferred from the surface. Full protonation of C_2_O_4_ to C_2_H_4_ requires the transfer of 12 electron/proton pairs. The product formation mechanism is predefined; thus, the selectivity of the process will not be determined, but instead we focus on the limiting steps and thermodynamic stability of the intermediates. We assume that each of the reaction steps may occur only through a particular intermediate; for instance, **20** can be converted to **26** by protonation of the O1 atom, but not to **24** as this would have required the rearrangement of bonds within the intermediate. This implies that the intermediates cannot freely interconvert within each row as shown in the Figure 2, and initially facile steps can lead to “dead ends” further down the process. On the other hand, we assume that the binding mode of the intermediate with respect to the surface might interchange. The corresponding free energy diagrams at U = 0 eV vs. RHE are illustrated together in Figure 4.

### 4.2. Pure Cu Catalyst

As was mentioned above, in this work, we have assumed that the formation of C−C bond takes place at the earliest stage of the process, by means of the formation of the oxalate anion (**1**) from two co-adsorbed CO_2_ molecules.

**1** is oriented horizontally, with the C−C bond aligned with the rows of Cu at the surface. As CO_2_ molecules are neutral in the gas phase, the formation of anionic **1** requires charge transfer to the reactive species, which can be achieved via the direct electron transfer from the cathode surface. This is confirmed by the DDEC charges—the total charge on the oxalate amounts to −0.73. At this stage, the C−C bond is relatively weak, with the order of 0.78, and the carbon atoms are slightly overcoordinated due to the interaction with the surface—the sum of bond orders (SBO) of each C atom amounts to approximately 4.12. This system is also characterized by a relatively strong bond between the oxygen atoms and the surface Cu atoms. The bond order with the closest Cu amounts to approx. 0.5 for each of the O atoms, and the distance is 1.98 Å.

The formation of anionic **1** is followed by its protonation to **2**. This step is exothermic by −0.09 eV. Due to the symmetry of this intermediate, either of the oxygen atoms can bind a proton. This leads to a change in the total charge on the intermediate, which now amounts to −0.28. It is caused by the binding of the proton, which now compensates part of the negative charge, but also by the charge of one of the carbon atoms, which increased to 0.52 from 0.46 with respect to **1**. The most stable configuration of **2** is horizontal, with the hydrogenated oxygen pointing away from the Cu surface.

The next step is the formation of oxalic acid (**3**). It is crucial, as it is a critical energetic step in the investigated pathway, and the calculated ΔG for this step amounts to 0.42 eV. Alternatively, **4** species can be formed; however, it is less stable by 0.09 eV. Two consecutive protonation steps for the same oxygen atom in **2** are highly undesirable, since **5** is even less stable—by as much as 0.48 eV with respect to **3**. Oxalic acid is oriented diagonally to the Cu rows, and remains parallel to the surface. This species is almost neutral, with two protons compensating the negative charge of the oxalate—the charge transferred is very small and amounts to −0.07. In addition, the C−C bond becomes stronger with the order of 1.023. This suggests the weakening of the interaction with the surface, which is indeed confirmed by the Cu−O bond order of 0.13 for the protonated O atoms; however, for the unprotonated oxygen atoms, the Cu−O bond order remains almost the same (0.49). This is also consistent with the Cu−O distances—2.04 Å and 2.74 Å for the deprotonated and protonated oxygen atoms, respectively. Subsequent (H^+^/e^−^) transfer leads to the formation of **7** species, which contains three hydroxyl groups. The most stable configuration is oriented horizontally, diagonally with respect to the Cu rows. This step does not involve any significant change in the charge transfer—the total charge of the intermediate is now −0.05 with respect to −0.07 for **3**. Interestingly, the positive charge of the proton is now compensated by a significantly lower charge of the C atom—0.14 with respect to 0.40 for **3**. The C−C bond order remains the same—1.02.

**Figure 5:**
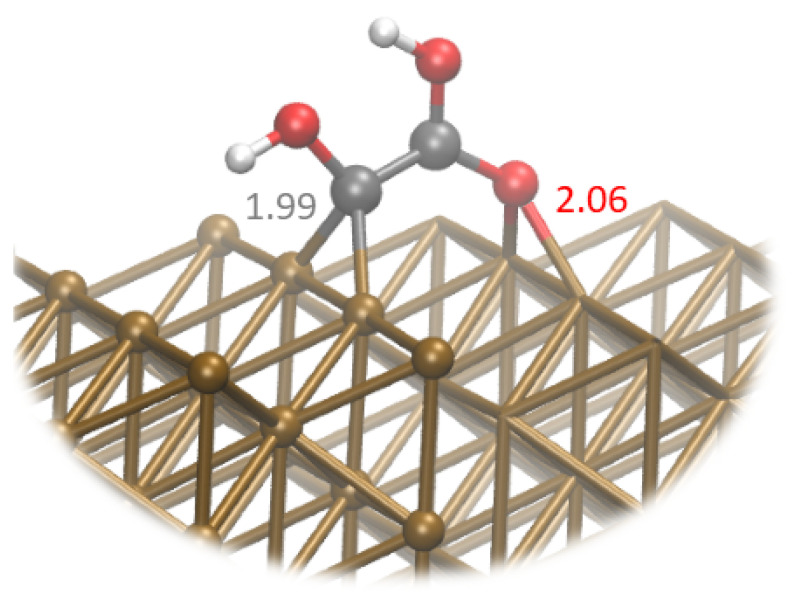
Snapshot of **12** species on pure Cu surface. Brown spheres represent Cu atoms, gray—C, white—H and red—O.

The next step is protonation of the hydroxyl group of **7** with the release of H_2_O. That leads to the formation of **12**, which is oriented perpendicularly to the surface. This geometry is characterized by the binding of C and O atoms across the Cu rows, where the carbon and the oxygen are coordinated to two Cu atoms. This is shown in Figure 5. In addition we observe the charge transfer from the copper surface to the intermediate, which becomes more negatively charged—−0.24. Interestingly, this coincides with the C atom bound to the surface becoming negatively charged—−0.24.

The formation of **18** is endothermic compared to the previous three steps (ΔG=0.21 eV). The optimization of the geometry leads to the intermediate being oriented perpendicularly to the surface, with one of the OH groups and the O atom interacting directly with the surface. The hydroxyl O atom is at the bridge site between two Cu atoms; the respective bond orders are 0.25 and 0.22. The lone O atom is also located at the bridge site; however, its interaction with the surface Cu atoms is much stronger—the bond orders are 0.46 and 0.47. Interestingly, the C−C bond in this intermediate is very strong with the order of 1.60. The resulting structure is the least stable intermediate in the path (ΔG=0.55 eV with respect to **1**).

Next, the protonation of the lone oxygen atom in **18** takes place, and the intermediate is transformed into **25**, which is again oriented parallel to the surface. As one of the C atoms is again within the range of the interaction with the surface, it forms a relatively strong bond with the Cu atom—the bond order is 0.49. This in turn weakens the C−C bond from the order of 1.60 to 1.24. Contrary to the previous transformation, this step is slightly exothermic (ΔG=−0.16 eV). In addition, the total charge increases from −0.17 for **18** to 0.20 for **25**.

Subsequently, H_2_O is released upon protonation of one of the hydroxyls, and **31** species is formed. This step is almost thermoneutral (ΔG=0.04 eV). The optimal geometry is the one with the perpendicular orientation, with the undercoordinated C atom interacting strongly with the surface at the bridge site. The C−Cu distances are 2.01 Å and the bond orders are 0.58 and 0.60. Due to the absence of oxygen, the C atom interacting with the surface gains a slight negative charge of −0.11. This is the step where the pathways for the pure Cu and Cu_2_Ni_6_ diverge, which will be explained in more detail further into the text.

The next step is followed by the conversion to **36**, which is characterized by two undercoordinated C atoms. Unsurprisingly, both C atoms tend to form a bond with the surface, and the preferred orientation is parallel to the surface. This is similar to **12**, in which the C−C bond is aligned parallel to the surface, and the interaction of both C atoms with the surface Cu atoms is significant. The carbons are at the bridge sites and the Cu−C distance is 2.07 Å and 2.02 Å for the −OH and −H bound carbon, respectively. The distances are consistent with bond orders—the longer and shorter bonds are of the order of 0.49 and 0.61, respectively. The bond character is also reflected in the charges borne by the C atoms—0.13 and −0.42 on the −OH and −H bound carbon, respectively. This step is again endothermic with ΔG of 0.09 eV.

Upon the protonation of the carbon atom in **36**, **40** species is formed. The most favorable geometry has the oxygen and carbon atoms coordinated to the surface Cu. Both the C and O atoms are located in the bridge sites, with the O−Cu distance of 2.44 Å being slightly longer than Cu−C, which amounts to 2.03 Å. In this case, the C atom interacts with the surface strongly due to the undercoordination, and the Cu−C bond order is 1.71 (compared to 0.22 for oxygen). This is consistent with significant stabilization of this intermediate—ΔG=−0.59 eV—with respect to **36**. The total charge becomes less negative and is q = −0.05 for **40** versus q = −0.15 for **36**.

The following step is exothermic again and yields **43** with ΔG=−0.35 eV. This species does not contain any undercoordinated atoms and the preferred orientation is horizontal with respect to the Cu surface. C atoms form only a weak bond with the surface, of the order 0.41; similarly, the O atom forms an even weaker bond—$BO_{Cu-O}$ = 0.15. This implies that the stabilization results from the interactions within the intermediate (ΔG=−0.42 eV with respect to **1**), and not from the interaction with the surface. In addition, the total charge increases from q = −0.05 for **40** to q = 0.22 for **43**.

The second-to-last step is the transformation to **44** with the energy increase of ΔG=0.33 eV. The intermediate is oriented vertically, with the singly hydrogenated C atom interacting strongly with Cu. The distance between the C atom and the closest Cu atom is 2.37 Å, and the respective bond order is 0.75.

The final product is ethylene (**45**), which is oriented horizontally with respect to the surface. It is the most stable (ΔG=−0.68 eV) in the entire pathway. An important observation is that the length of the C−C bond is 1.39 Å, different from the value of 1.33 Å, found in the gas phase. This corresponds to the bond order of 1.51, which is nominally weaker due to the non-negligible interaction with the surface.

### 4.3. Cu-Ni Bimetallic Surfaces

In this section, we discuss the differences in the properties of the bimetallic surfaces consisting of a single and a double Cu overlayer deposited on the Ni surface with respect to the clean Cu surface. The solid green and blue lines in Figure 2 show the energy profiles for the preferred pathways for a single Cu overlayer denoted as Cu_1_Ni_7_ (pathway II) and double overlayer denoted as Cu_2_Ni_6_ (pathway III), respectively.

All reactions proceed similarly through the first six steps, with differences only in the binding modes and interaction energies, but not in the intermediates involved. As mentioned in the previous section, the formation of anionic **1** requires a charge transfer from the metal surface. The total charge on the intermediate is only slightly more negative and amounts to q = −0.76 and q = −0.73 for the single and dual layer, respectively compared to q = −0.73 for the case of a pure Cu surface.

The first transfer of the electron/proton pair is similar to a pure Cu surface. In the case of Cu_1_Ni_7_, the step is still exothermic by −0.17 eV, but in the case of Cu_2_Ni_6_, the process is thermoneutral (ΔG of 0.03 eV is negligible). In both bimetallic systems, the change in the total charge on the intermediate is noticeable, and the systems become less negatively charged by approximately 0.4 with respect to **1**. This is consistent with a proton being bound to one of the oxygen atoms to compensate the charge. The preferred geometry of the **2** species is similar to that for a pure Cu surface.

Transformation of **2** → **3** is a critical energetic step for the Cu_2_Ni_6_ surface. The formation of oxalic acid (**3**) is the most endothermic step and requires as much as 0.49 eV, which is even more than in the case of pure Cu surface—and therefore it is a limiting step. Despite the similar configuration of **3**, this species bears more negative charge of q = −0.14, compared to q = −0.07 for pure Cu surface. The C−C bond order equals to 1.11 and is slightly stronger compared to the **3** species in other systems.

**Figure 6:**
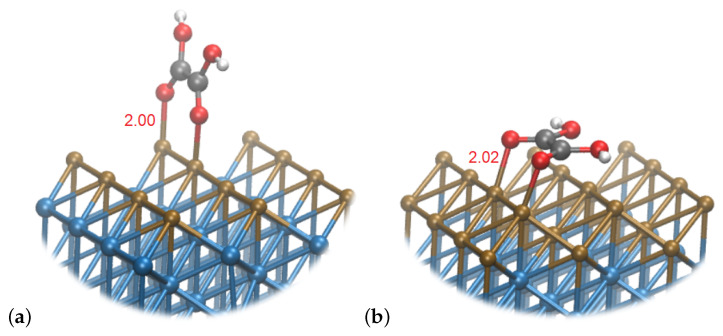
Snapshot of **3** species on (**a**) Cu_1_Ni_7_ and (**b**) Cu_2_Ni_6_. Brown spheres represent Cu atoms, blue—Ni, gray—C, white—H and red—O.

Interestingly, the formation of **3** at the Cu monolayer surface requires only 0.12 eV, and it is not a limiting step in the process, contrary to both pure Cu and Cu_2_Ni_6_ surfaces. This is related to a different preferred configuration, where the plane of the molecule is oriented perpendicularly to the surface, with the C−C bond parallel to the surface, aligned with the Cu rows. This is shown in Figure 6. The interaction of deprotonated oxygen atoms with the Cu layer slightly compensates for the negative charge—the charges on both O atoms amount to −0.41, compared to −0.42 in the case of the same intermediate bound to Cu_2_Ni_6_. In both cases, the total charge of the intermediate is 0.04, implying its neutral state and covalent binding to the surface.

The formation of intermediate **7** with three hydroxyl groups is a slightly exothermic process for the Cu_1_Ni_7_ system, where ΔG amounts to −0.29 eV. The charge transfer involved in this step is insignificant: −0.06 versus −0.04 for **3**. On the other hand, a significant change in charge transfer has been observed in the Cu_2_Ni_6_ system—the total charge of **7** is −0.04 with respect to −0.14 for **3**.

Subsequent hydrogenation leads to the formation of **12** with the release of H_2_O. In the cases of a single Cu surface, this step is slightly endothermic with the ΔG of 0.15 eV, but is thermoneutral for a double Cu layer system (ΔG = 0.06 eV). For comparison, in the case of pure Cu surface this step is also thermoneutral; ΔG is below the accuracy of the method used.

Species **18** differ with respect to the preferred configurations on different surfaces. On Cu_1_Ni_7_, the intermediate is oriented perpendicularly to the surface, as in the pure Cu system; however, the two cases differ in the orientation relative to Cu rows. At the Cu_1_Ni_7_ surface, **18** lays across and on the pure Cu intermediate **18** is aligned with the Cu rows. In both instances, the configuration is the least stable among the other intermediates in the entire pathways (on Cu_1_Ni_7_ ΔG=0.47 eV with respect to **1**). On Cu_2_Ni_6_, 18 is oriented perpendicularly to the surface, with the C−C bond being oriented parallel to the surface and aligned to Cu rows.

On all investigated surfaces, the 18 intermediate is characterized by a strong C−C bond and its order is 1.47 and 1.56 for the Ni surface with single and double Cu layers, respectively. This is consistent with the strong C−C bond observed for pure Cu surface of the order of 1.56.

The formation of **18** is almost thermoneutral with ΔG equal to −0.01 eV and 0.09 eV for the double and single Cu layer, respectively. In comparison, for the pure Cu surface, the transformation is slightly endothermic with ΔG amounting to 0.21 eV.

The formation of **25** with three hydroxyl groups is similar for the investigated surfaces. This conversion is exothermic for all surfaces, ΔG amounts to −0.32 eV and −0.51 eV for Cu_1_Ni_7_ and Cu_2_Ni_6_. The total charge of **25** becomes positive, and the values are q = 0.19 and q = 0.21 for the systems of single and double Cu layer, respectively.

The protonation of **25** is where the pathways diverge and different intermediates become the most stable for the different investigated surfaces. On the Cu_1_Ni_7_ it is **31**, which is the same as in the pure Cu system. It is similarly oriented, with the undercoordinated site of the carbon atom bound to the hydroxyl group interacting with the surface. This step is slightly more endothermic than on a pure Cu surface; ΔG amounts to 0.16 eV. Despite this, no significant differences in bond orders or total charges are observed.

**Figure 7:**
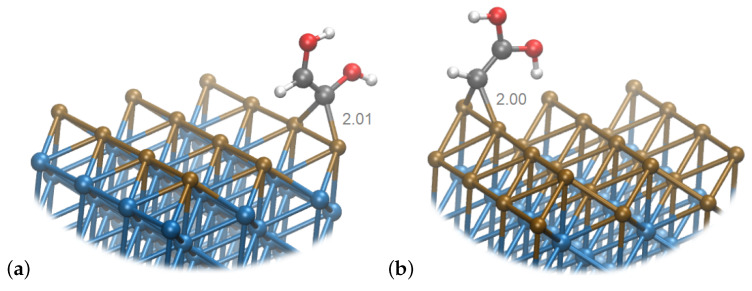
Snapshot of (**a**) **31** species on Cu_1_Ni_7_ and (**b**) **32** species on Cu_2_Ni_6_. Brown spheres represent Cu atoms, blue—Ni, gray—C, white—H and red—O.

Contrary to this, in Cu_2_Ni_6_, the **32**, in which two hydroxyl groups are bound to the same C atom, is thermodynamically preferred. The intermediate is oriented perpendicularly to the surface, and the undercoordinated C atom interacts with the surface. This transformation is also characterized by the greatest change in the charge: on Cu_2_Ni_6_—the charge of the intermediate changes to q = −0.12 from q = 0.21 for **25**. The transformation is endothermic with ΔG=0.31 eV, and the C−C bond becomes stronger, with the bond order of 1.48 for **32** on the Cu_2_Ni_6_ system. The geometries and the binding modes of these intermediates on bimetallic surfaces are shown in Figure 7.

The next two protonation steps are very similar on pure Cu and Cu_1_Ni_7_ with binding modes, interaction energies and free energy profiles. Similarly to the pure Cu surface, in **40**, the bond C−C becomes significantly stronger and its order amounts to 1.73 compared to 1.48 for **36**. In both cases, **40** has the strongest C−C bond among the investigated intermediates.

On the Ni surface with a double Cu layer, the transformation is different. The undercoordinated C atom is protonated and forms 38. Upon the binding of hydrogen, the total amount of charge bore by the intermediate increases to q = 0.236. This transformation is exothermic (ΔG=−0.31 eV), which makes it different from endothermic processes on other surfaces. This configuration is characterized by a slightly smaller C−C bond order—1.409 compared to 1.485 for **32**.

The next step is the protonation of the hydroxyl group of **38** with the release of H_2_O, which leads to the formation of **41**. It is oriented parallel to the surface, diagonally with respect to the Cu rows. Compared to the previous transformation, this step is endothermic by 0.38 eV. The total charge on the intermediate becomes slightly negative and amounts to q = −0.04 for **41**. This is also characterized by a stronger C−C bond order—1.52.

**Figure 8:**
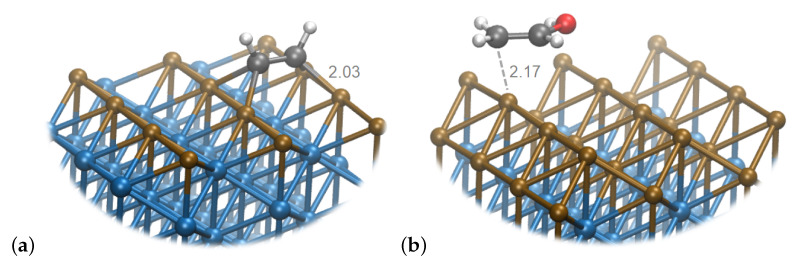
Snapshot of (**a**) **42** species on Cu_1_Ni_7_ and (**b**) **43** species on Cu_2_Ni_6_. Brown spheres represent Cu atoms, blue—Ni, gray—C, white—H and red—O.

The following transformation is converging the paths at pure Cu with the Cu_2_Ni_6_ systems. The same intermediate (**43**) is formed, with analogous configuration on both surfaces (see Figure 8). Also, similarly to the pure Cu, the transformation is exothermic and ΔG is −0.51 eV (compared to −0.35 eV on pure Cu). Additionally, the C−C bond is of the same order, as in the previous step, and amounts to 1.52.

For the Cu_1_Ni_7_ system, the equivalent step involves protonation of the hydroxyl group of **40** with the release of H_2_O, which is still different than on the other systems, and the paths still do not converge. The formation of the **42** species is endothermic by 0.41 eV and this is the limiting step for entire pathway. This is important, because in the case of other systems, the limiting steps were in the initial stage of the process. Additionally, the potential limiting step is formally the smallest among the investigated system, but the difference with respect to the pure Cu system is below the accuracy of the method used.

The reason for the endothermicity of this step is the large negative total charge which needs to be transferred to the intermediate—q = −0.22. The order of the C−C bond is slightly smaller and amounts to 1.66 compared to 1.73 for **40**. The intermediate formed in this step is oriented perpendicularly to the surface, with both hydrogenated C atoms interacting with the Cu monolayer.

The following step leads to the convergence of all the paths, in which the same intermediate is formed—**44**. The transformation is endothermic for the Cu_2_Ni_6_, similarly to the pure Cu surface; ΔG in both instances is 0.33 eV. In both cases, the C−C bond becomes stronger, and for Cu_2_Ni_6_ is the strongest for the entire pathway (C−C bond order is 1.67).

Contrary to that, on the Cu_1_Ni_7_ surface, the analogous step is exothermic by ΔG = −0.45 eV. In general, in all cases **44** is oriented perpendicularly to the surface and the undercoordinated C atom interacts with Cu. The C−C bond is slightly stronger and its order amounts to 1.70 versus 1.657 for **42**.

The final step of the mechanism is the same in all investigated systems, and leads to the formation of C_2_H_4_. It is the most thermodynamically stable compound with a length of the C−C bond as short as 1.39 Å and of the order of 1.52 and 1.51 for the Ni surface with a single and double Cu layer, respectively. The strongest interaction of ethylene with the surface is observed for Cu_1_Ni_7_—ΔG is 1.02 eV. In both bimetallic systems, the total charge is very close to q = 0.1. The smallest total charge and slightly weaker interaction with the surface is observed on Cu pure (q = 0.09).

## 5. Interactions with the Surface

The investigated pathways differ with respect to the potential energy profiles, which is the result of the surface composition. It needs to be stressed that the intermediates interact directly with the Cu atoms of the surface in each case. Despite this, we have observed significant differences in the characteristics of the systems, ranging from the difference in the free energy and different binding modes to the different most stable intermediate.

The free energy profile implies that the Ni surface with one Cu overlayer has the most favorable electrocatalytic properties with the potential limiting step of η = 0.41 eV; however, the difference with respect to the other systems is practically negligible. The potential limiting step for this pathway is the transformation **40** → **42**, which is associated with the formation of negatively charged intermediate (q = −0.222). This is opposite to the pure Cu surface and Cu_2_Ni_6_ systems, in which the most stable intermediate is **43** bearing a positive charge of q = 0.218 and q = 0.229, respectively. A similar effect has been observed for the intermediate **38**, which is most stable at the Cu_2_Ni_6_ surface, and **36**—most stable at pure Cu and Cu_1_Ni_7_. **38** bears a positive charge of q = 0.236, compared to **36**—bearing a negative charge of q = −0.153 and q = −0.150 for the pure Cu and Cu_1_Ni_7_, respectively. Both these observations imply that there is a significant role for the charge transfer, which is responsible for the stability of the particular intermediates.

**Figure 9:**
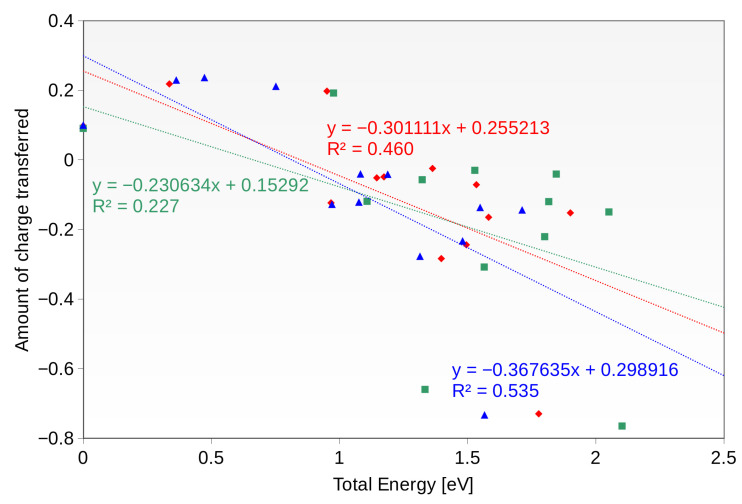
The correlation of the potential energy of the system with the charge transferred to the intermediates.

Figure 9 shows the correlation between the amount of the charge transferred and the potential energy of the particular intermediate. For this analysis, only the potential energy has been considered as calculated by VASP software, for compatibility with the DDEC6 charges calculation scheme—thus, the correlations are evaluated without the ZPE corrections or the solvation effect.

The correlations for each investigated system are far from perfect, and in the best case the $R^{2}$ coefficient is slightly greater than 0.5. This implies that there is another factor playing an important role in the stabilization of the intermediates. On the other hand, it is worth noting that all of the systems in which the amount of the charge transferred is greater than q = 0.5 are characterized by a relatively low stability compared to the most stable system. This, on the other hand, implies that the charge transfer is at least partially responsible for the observed effect.

**Figure 10:**
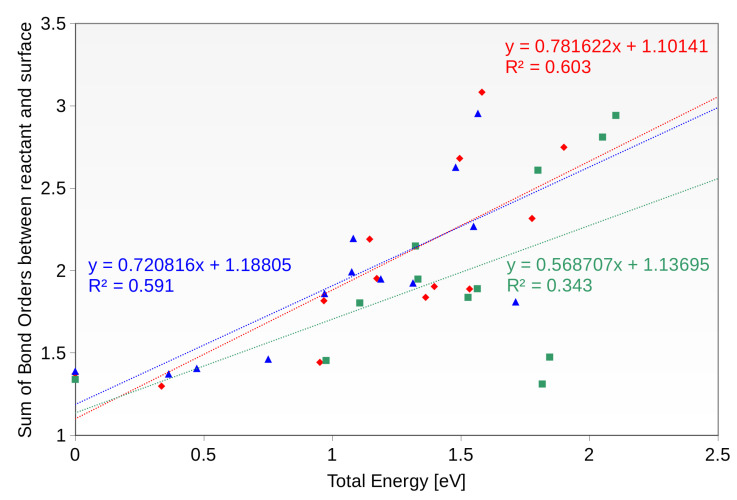
The correlation of the potential energy of the system with the sum of all bond orders between the intermediate and the surface.

The other factor that might play a role is the strength of the covalent bonds between the intermediate and the surface. As was mentioned before, all the intermediates are directly bound to the Cu layer in each investigated case. Surprisingly, we have observed significant differences between investigated surfaces (see Figure 10). Similarly to the correlation with the charge transferred, the system with one copper layer is characterized by the smallest correlation coefficient ($R^{2}$ = 0.34). The other systems—pure Cu and Ni with 2 Cu layers—are characterized by greater $R^{2}$ values of approximately 0.6.

We conclude that both of the factors—the amount of charge transferred and the covalent bonding to the surface—contribute to the final potential energy of the system. Interestingly, the system with one Cu layer does not show a significant correlation with respect to either of these factors. That suggests the presence of the other factors, we have not been able to identify, or a combination of those.

## 6. Discussion

The results indicate that the underlying Ni is able to significantly influence the interactions with the Cu surface. Despite the fact that the intermediates interact directly with the Cu atoms in all cases, the predicted mechanisms are different. Compared with the pure Cu surface, we have observed that at other surfaces, different intermediates become the most stable—one difference in the case of one Cu layer in Ni, and three in the case of two Cu layers. In addition to this, differences in binding modes have also been observed. This suggests that there is another factor responsible for the stabilizing effect, other than the covalent bonds between the intermediates and the surface.

The reaction potential is affected only to a small degree—η=0.42 V for the clean Cu surface, for 1 and 2 Cu overlayers on Ni η=0.42 V and η=0.48 V, respectively. However, binding modes, and the resulting binding energy of the intermediates, are affected to a greater degree. As Ni and Cu have almost identical lattice constants, the geometrical factor cannot be considered responsible for these differences, and the reason should be looked for in the electronic structure of these materials. This is confirmed by DDEC6 charges, which indicate differences in the amount of the charge transferred to the intermediates.

This is best shown in the example of the **18** → **25** transformation: **18** is oriented perpendicularly to the surfaces of pure Cu and Cu_1_Ni_7_, and parallel in the case of Cu_2_Ni_6_. Interestingly, despite the differences in geometry, the stability of this intermediate is very similar in all cases (see Figure 4). The formation of **25** gives rise to a positively charged intermediate, which in all cases is geometrically similar (oriented parallel to the surface), but is significantly more stable on the Cu_2_Ni_6_ system.

Most importantly, however, the effect of Ni is visible in the different intermediates becoming more stable on different surfaces. This affects the later stages of the process in particular, where the limiting step for the Cu_1_Ni_7_ system has been observed. The consensus of the current understanding of the process of CO_2_ reduction is that the C−C bond is formed at the later stage of the process, which is consistent with our simulations. The potential limiting steps we have observed took place in the early stages, and therefore it is very likely that there are other, more preferred pathways leading to the final product. However, the effect of the Ni on the interactions between the reactants and the Cu surface is significant enough to push the potential limiting step towards the further stages of the process. Therefore, the possibility of the formation of the C−C bond at the very first step cannot be ruled out.

## 7. Conclusions

The C−C bond formation is a key step towards the C_2_^+^ products in the electrocatalytic CO_2_ conversion. In this study, we have used the DFT calculations to investigate the effect of alloying Cu and Ni on the electrocatalytic activity for the reduction of CO_2_ to C_2_H_4_ and compared it to results with those obtained for the pure Cu surface.

DFT calculations predict that the formation of C_2_H_4_ from C_2_O_4_ is possible on the Cu surface with the applied potential of about 0.42 eV. This is consistent with the results in the literature [31], despite the differences in the mechanism assumed in this work. The potential limiting step occurs in the early stage of the process, with the transformation **2** → **3**, suggesting the route where the C−C bond is formed at the later stage might indeed be preferred.

The addition of Ni causes a synergistic effect: one overlayer of Cu on top of the Ni surface causes a slight decrease in the potential, but more importantly shifts the limiting step towards the end of the path, to the **40** → **42** transformation. Interestingly, the addition of a second Cu overlayer on the same Ni surface increases the limiting potential by 0.08 eV.

## Data Availability

Not applicable.

## Supplementary Materials

The following supporting information can be downloaded at: https://www.mdpi.com/article/10.3390/ma16145138/s1.

## References

[1] Maginn E.J. (2010). What to Do with CO_2_. J. Phys. Chem. Lett..

[2] Zhu Q. (2019). Developments on CO_2_-utilization technologies. Clean Energy.

[3] Alper E., Yuksel Orhan O. (2017). CO_2_ utilization: Developments in conversion processes. Petroleum.

[4] Li Y., Hwa Chan S., Sun Q. (2015). Heterogeneous catalytic conversion of CO_2_: A comprehensive theoretical review. Nanoscale.

[5] Parigi D., Giglio E., Soto A., Santarelli M. (2019). Power-to-fuels through carbon dioxide Re-Utilization and high-temperature electrolysis: A technical and economical comparison between synthetic methanol and methane. J. Clean. Prod..

[6] Palys M.J., Daoutidis P. (2022). Power-to-X: A review and perspective. Comput. Chem. Eng..

[7] Ran J., Jaroniec M., Qiao S.Z. (2018). Cocatalysts in Semiconductor-based Photocatalytic CO_2_ Reduction: Achievements, Challenges, and Opportunities. Adv. Mater..

[8] Owusu P.A., Asumadu-Sarkodie S. (2016). A review of renewable energy sources, sustainability issues and climate change mitigation. Cogent Eng..

[9] Hori Y. (2008). Electrochemical CO_2_ Reduction on Metal Electrodes. Modern Aspects of Electrochemistry.

[10] Nitopi S., Bertheussen E., Scott S.B., Liu X., Engstfeld A.K., Horch S., Seger B., Stephens I.E., Chan K., Hahn C. (2019). Progress and Perspectives of Electrochemical CO_2_ Reduction on Copper in Aqueous Electrolyte. Chem. Rev..

[11] Bagger A., Ju W., Varela A.S., Strasser P., Rossmeisl J. (2017). Electrochemical CO_2_ Reduction: A Classification Problem. ChemPhysChem.

[12] Sharifian R., Wagterveld R.M., Digdaya I.A., Xiang C., Vermaas D.A. (2021). Electrochemical carbon dioxide capture to close the carbon cycle. Energy Environ. Sci..

[13] Tan X., Yu C., Ren Y., Cui S., Li W., Qiu J. (2021). Recent advances in innovative strategies for the CO_2_ electroreduction reaction. Energy Environ. Sci..

[14] Zhang S., Fan Q., Xia R., Meyer T.J. (2020). CO_2_ Reduction: From Homogeneous to Heterogeneous Electrocatalysis. Accounts Chem. Res..

[15] Bu Y.F., Zhao M., Zhang G.X., Zhang X., Gao W., Jiang Q. (2019). Electroreduction of CO_2_ on Cu Clusters: The Effects of Size, Symmetry, and Temperature. ChemElectroChem.

[16] Dong H., Li Y., Jiang D.E. (2018). First-Principles Insight into Electrocatalytic Reduction of CO_2_ to CH4 on a Copper Nanoparticle. J. Phys. Chem. C.

[17] Xu H., Rebollar D., He H., Chong L., Liu Y., Liu C., Sun C.J., Li T., Muntean J.V., Winans R.E. (2020). Highly selective electrocatalytic CO_2_ reduction to ethanol by metallic clusters dynamically formed from atomically dispersed copper. Nat. Energy.

[18] He J., Johnson N.J.J., Huang A., Berlinguette C.P. (2018). Electrocatalytic Alloys for CO_2_ Reduction. ChemSusChem.

[19] Kim D., Resasco J., Yu Y., Asiri A.M., Yang P. (2014). Synergistic geometric and electronic effects for electrochemical reduction of carbon dioxide using gold–copper bimetallic nanoparticles. Nat. Commun..

[20] Li M., Wang J., Li P., Chang K., Li C., Wang T., Jiang B., Zhang H., Liu H., Yamauchi Y. (2016). Mesoporous palladium–copper bimetallic electrodes for selective electrocatalytic reduction of aqueous CO_2_ to CO. J. Mater. Chem. A.

[21] Minati L., Speranza G., Calliari L., Micheli V., Baranov A., Fanchenko S. (2008). The Influence of Metal Nanoparticle Size Distribution in Photoelectron Spectroscopy. J. Phys. Chem. A.

[22] Siegel J., Kvítek O., Ulbrich P., Kolská Z., Slepička P., Švorčík V. (2012). Progressive approach for metal nanoparticle synthesis. Mater. Lett..

[23] Sharma G., Kumar A., Sharma S., Naushad M., Prakash Dwivedi R., ALOthman Z.A., Mola G.T. (2019). Novel development of nanoparticles to bimetallic nanoparticles and their composites: A review. J. King Saud Univ.-Sci..

[24] Kuhl K.P., Cave E.R., Abram D.N., Jaramillo T.F. (2012). New insights into the electrochemical reduction of carbon dioxide on metallic copper surfaces. Energy Environ. Sci..

[25] Klier K. (1982). Methanol Synthesis. Advances in Catalysis.

[26] Peterson A.A., Abild-Pedersen F., Studt F., Rossmeisl J., Nørskov J.K. (2010). How copper catalyzes the electroreduction of carbon dioxide into hydrocarbon fuels. Energy Environ. Sci..

[27] Fan L., Xia C., Yang F., Wang J., Wang H., Lu Y. (2020). Strategies in catalysts and electrolyzer design for electrochemical CO_2_ reduction toward C_2_ products. Sci. Adv..

[28] Zhou Y., Yeo B.S. (2020). Formation of C–C bonds during electrocatalytic CO_2_ reduction on non-copper electrodes. J. Mater. Chem. A.

[29] Zhu W., Tackett B.M., Chen J.G., Jiao F. (2018). Bimetallic Electrocatalysts for CO_2_ Reduction. Top. Curr. Chem..

[30] Ruiz-López E., Gandara-Loe J., Baena-Moreno F., Reina T.R., Odriozola J.A. (2022). Electrocatalytic CO_2_ conversion to C_2_ products: Catalysts design, market perspectives and techno-economic aspects. Renew. Sustain. Energy Rev..

[31] Calle-Vallejo F., Koper M.T.M. (2013). Theoretical Considerations on the Electroreduction of CO to C_2_ Species on Cu(100) Electrodes. Angew. Chem. Int. Ed..

[32] Kresse G., Furthmüller J. (1996). Efficient iterative schemes for ab initio total-energy calculations using a plane-wave basis set. Phys. Rev. B-Condens. Matter Mater. Phys..

[33] Kresse G., Joubert D. (1999). From ultrasoft pseudopotentials to the projector augmented-wave method. Phys. Rev. B-Condens. Matter Mater. Phys..

[34] Perdew J.P., Burke K., Ernzerhof M. (1996). Generalized gradient approximation made simple. Phys. Rev. Lett..

[35] Blöchl P.E. (1994). Projector augmented-wave method. Phys. Rev. B.

[36] Monkhorst H.J., Pack J.D. (1976). Special points for Brillouin-zone integrations. Phys. Rev. B.

[37] VTSTTools 3.1.

[38] Mathew K., Sundararaman R., Letchworth-Weaver K., Arias T.A., Hennig R.G. (2014). Implicit solvation model for density-functional study of nanocrystal surfaces and reaction pathways. J. Chem. Phys..

[39] Manz T.A., Limas N.G. (2016). Introducing DDEC6 atomic population analysis: Part 1. Charge partitioning theory and methodology. RSC Adv..

[40] Limas N.G., Manz T.A. (2016). Introducing DDEC6 atomic population analysis: Part 2. Computed results for a wide range of periodic and nonperiodic materials. RSC Adv..

[41] Manz T.A. (2017). Introducing DDEC6 atomic population analysis: Part 3. Comprehensive method to compute bond orders. RSC Adv..

