# Supplementary material for: The Influence of Ni Addition in the Mechanism of CO2 Electroreduction on Cu Crystals—Mechanistic Insight from DFT Simulations
Source: Materials (Basel). 2023 Jul 21;16(14):5138. doi: 10.3390/ma16145138 (PMC10384679; doi:10.3390/ma16145138)
Supplement: Supplementary file 1 [file materials-16-05138-s001.zip › materials-2489250-supplementary.pdf]

Supporting Information

The Influence of Ni Addition in the Mechanism of CO_2 Electroreduction on Cu Crystals—Mechanistic Insight from DFT Simulations

Elżbieta Dziadyk-Stopyra [1], Ionut Tranca [2], Daniel Smykowski [3] and Bartłomiej M. Szyja [1,*]

[1] Institute of Advanced Materials, Faculty of Chemistry, Wrocław University of Science and Technology, Gdańska 7/9, 50-344 Wrocław, Poland; elzbieta.dziadyk@pwr.edu.pl
[2] Algemene Chemie, Vrije Universiteit Brussel, Pleinlaan 2, B-1050 Brussels, Belgium; ionut.tranca@vub.be
[3] Department of Energy Conversion Engineering, Faculty of Mechanical and Power Engineering, Wrocław University of Science and Technology, Wybrzeże Wyspiańskiego 27, 50-370 Wrocław, Poland; daniel.smykowski@pwr.edu.pl
* Correspondence: b.m.szyja@pwr.edu.pl; Tel.: +48-71-320-6370

Contents

S1 Structures of the optimized intermediates 2
S2 Energy components of the intermediates 5
S3 Partial charges on the atoms of the intermediates 8
S4 Sum of Bond Orders 9

S1 Structures of the optimized intermediates

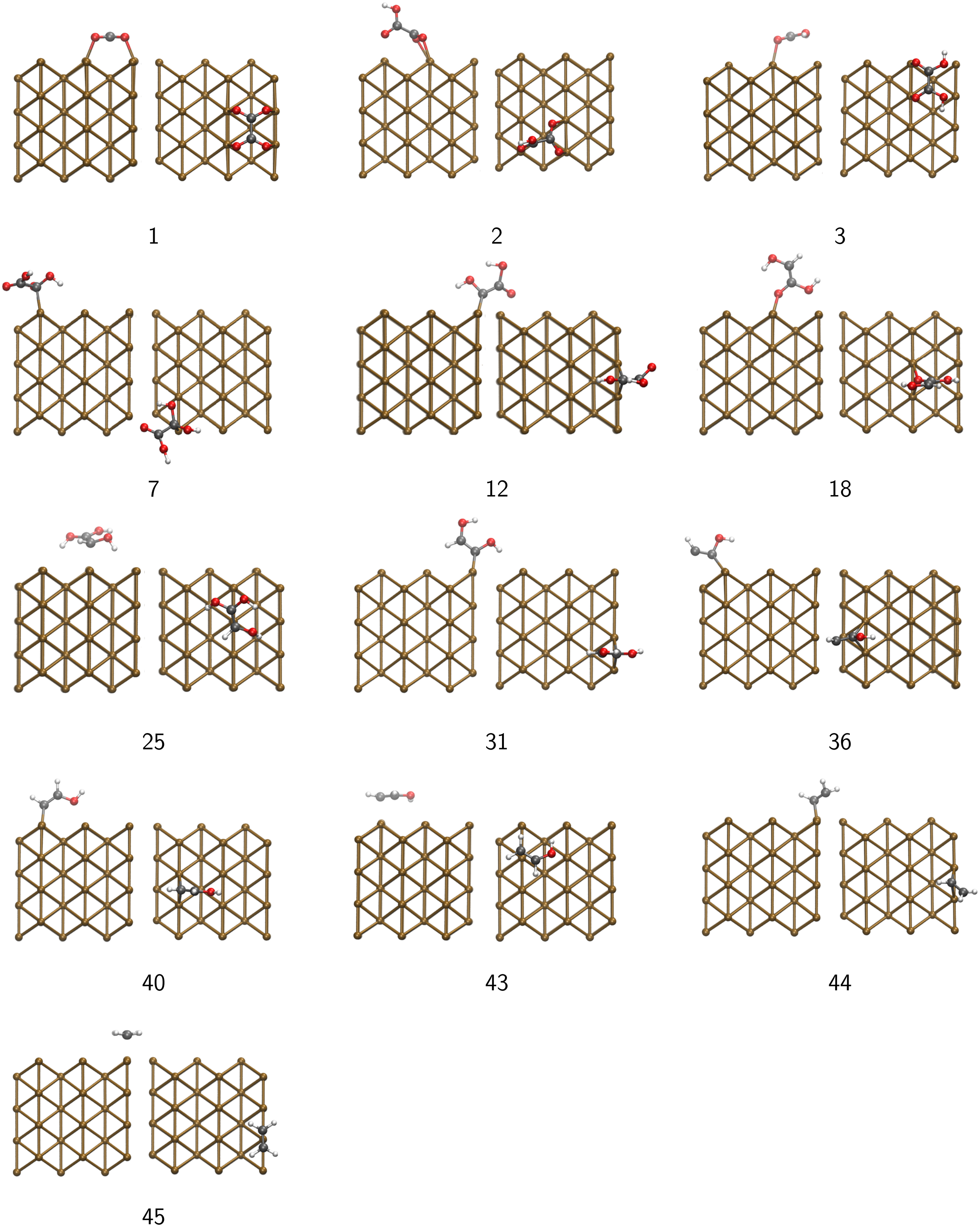

Figure S1: Side and top views of structures of the optimized intermediates on a pure Cu surface.

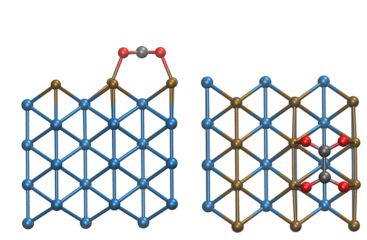

1

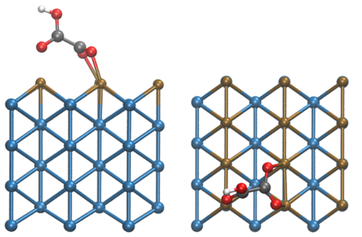

2

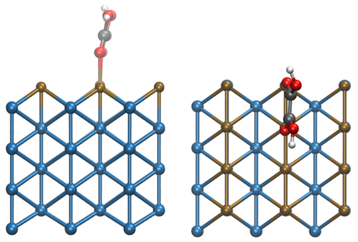

3

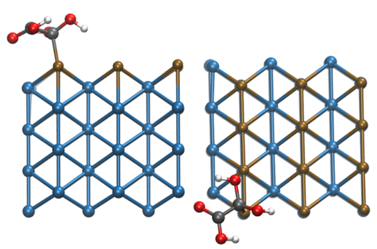

7

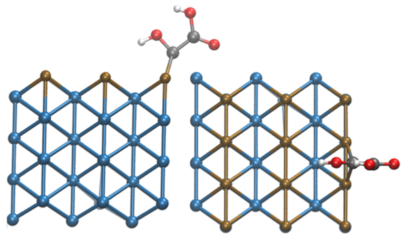

12

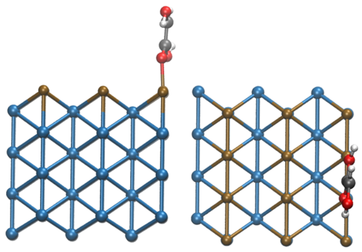

18

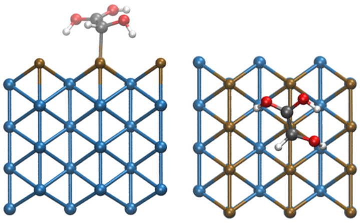

25

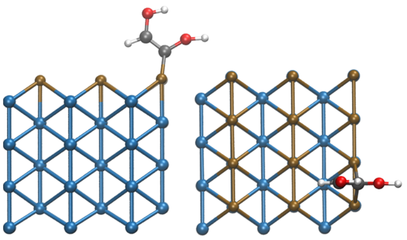

31

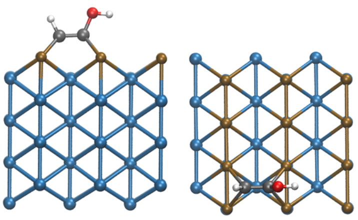

36

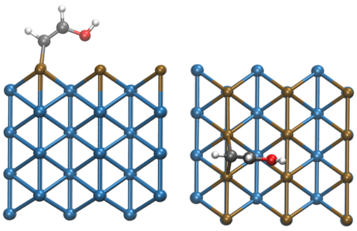

40

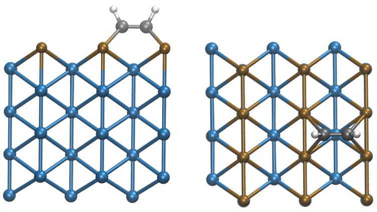

42

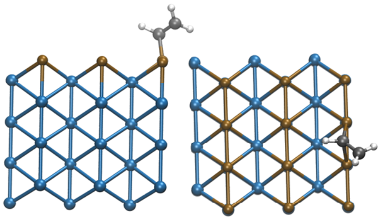

44

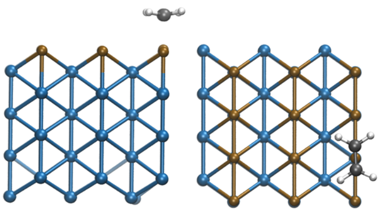

45

Figure S2: Side and top views of structures of the optimized intermediates on the 1 overlayer Cu surface

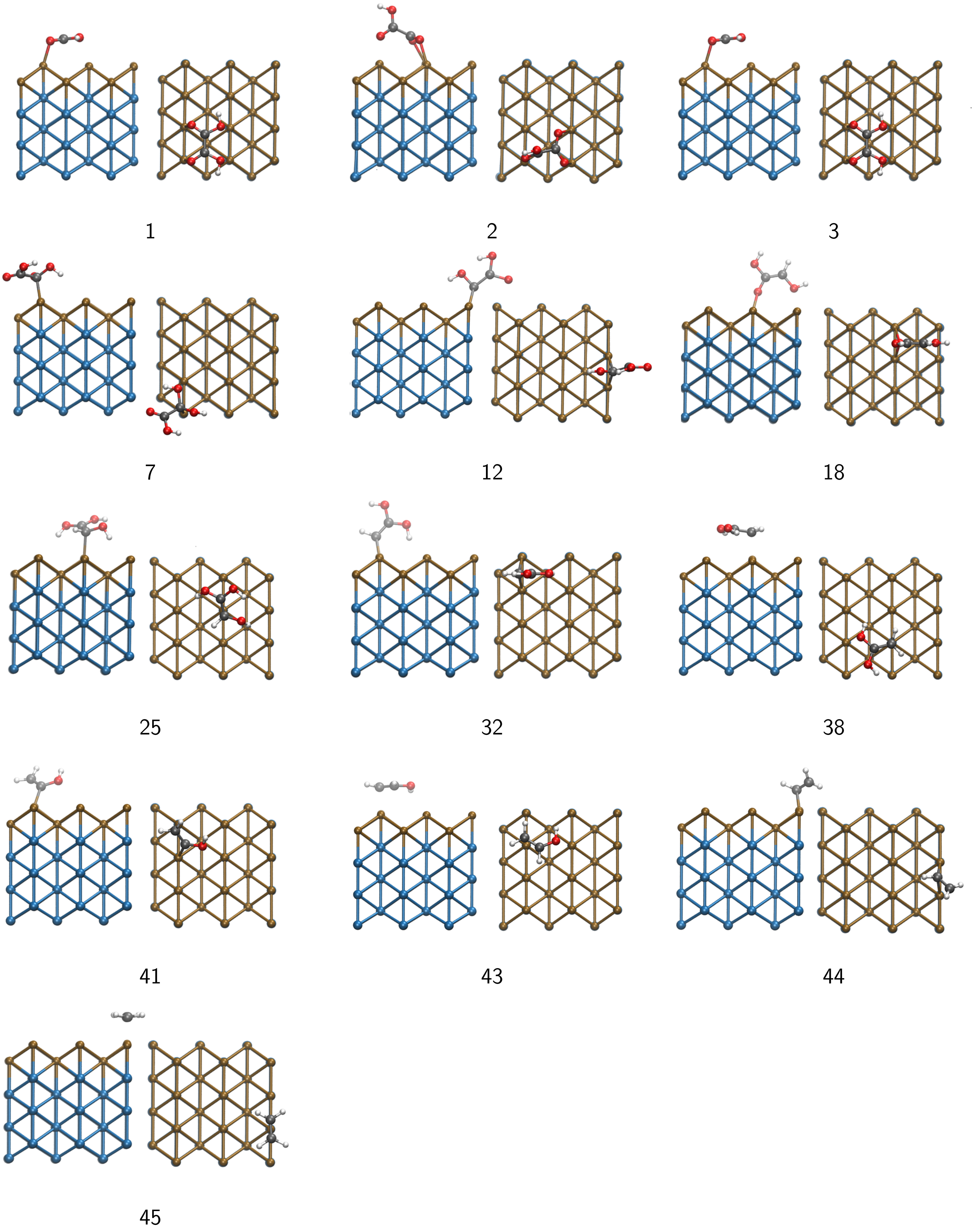

Figure S3: Side and top views of structures of the optimized intermediates on the 2 overlayers Cu surface

S2 Energy components of the intermediates

Table S1: Energy components of the optimized intermediates on the pure Cu surface

System	DFT Energy	ZPE	Solvation	System	DFT Energy	ZPE	Solvatation
1	−427.91	3.72	−428.24	31	−423.69	4.40	−423.85
2	−431.53	4.05	−431.90	32	−424.01	4.40	−424.28
3	−434.64	4.31	−434.97	33	−424.94	4.39	−425.11
4	−434.56	4.29	−434.88	34	−413.66	3.97	−413.69
5	−419.95	3.59	−420.12	35	−427.63	4.52	−427.82
6	−423.48	3.87	−423.64	36	−411.91	3.95	−412.89
7	−438.26	4.63	−438.54	37	−413.26	3.95	−413.30
8	−423.48	3.89	−423.65	38	−427.89	4.68	−428.12
9	n/a	n/a	n/a	39	−417.63	4.27	−417.67
10	−412.88	3.41	−412.91	40	−416.70	4.27	−417.04
11	−427.50	4.18	−427.79	41	−416.67	4.26	−416.84
12	−427.50	4.21	−427.69	42	−405.65	3.80	−405.65
13	−427.83	4.19	−428.05	43	−420.78	4.57	−420.93
14	−440.68	4.93	−441.44	44	−409.73	4.13	−409.75
15	−427.15	4.22	−427.30	45	−413.94	4.45	−413.99
16	−415.85	3.74	−415.93				
17	−415.79	3.74	−416.00				
18	n/a	n/a	n/a				
19	−430.71	4.48	−430.89				
20	−416.40	3.77	−416.87				
21	−430.65	4.51	−431.02				
22	−419.92	4.03	−420.06				
23	−420.19	4.05	−420.32				
24	−419.84	4.07	−420.00				
25	−434.52	4.80	−434.70				
26	−420.80	4.11	−421.04				
27	−421.38	4.10	−421.49				
28	−420.36	4.09	−419.88				
29	−424.31	4.37	−424.39				
30	−409.56	3.65	−409.61				

Table S2: Energy components of the optimized intermediates on the 1 overlayer Cu surface

System	DFT Energy	ZPE	Solvation	System	DFT Energy	ZPE	Solvation
1	−560.17	4.05	−560.59	31	−556.10	4.72	−556.74
2	−563.95	4.39	−564.34	32	−556.43	4.73	−556.74
3	−566.90	4.46	−567.53	33	−557.32	4.72	−557.55
4	−566.96	4.62	−567.32	34	−545.92	4.29	−546.00
5	−552.34	3.91	−552.61	35	−560.10	5.02	−560.29
6	−555.96	4.23	−556.16	36	−545.16	4.27	−545.35
7	−570.67	4.97	−570.99	37	−545.61	4.28	−545.71
8	−556.07	4.25	−556.26	38	−560.40	5.01	−560.68
9	n/a	n/a	n/a	39	−550.03	4.60	−550.11
10	−545.54	3.76	−545.62	40	−549.12	4.60	−549.49
11	−559.84	4.53	−560.20	41	−549.09	4.59	−549.31
12	−559.77	4.54	−560.00	42	−538.14	4.13	−538.19
13	−560.10	4.83	−560.35	43	−553.35	4.90	−553.53
14	−573.20	5.26	−574.01	44	−542.17	4.48	−542.23
15	−560.10	4.83	−560.35	45	−546.52	4.80	−546.60
16	−547.93	4.08	−548.18				
17	−548.22	4.09	−548.44				
18	n/a	n/a	n/a				
19	−563.15	4.82	−563.36				
20	−548.64	4.74	−549.14				
21	−562.99	4.83	−563.43				
22	−552.34	4.37	−552.52				
23	−552.45	4.39	−552.62				
24	−552.29	4.41	−552.50				
25	−567.07	5.13	−567.30				
26	−553.05	4.44	−553.33				
27	−553.65	4.43	−553.81				
28	−552.03	4.41	−552.30				
29	−556.69	4.70	−556.82				
30	−541.95	3.99	−542.04				

Table S3: Energy components of the optimized intermediates on the 2 overlayers Cu surface

System	DFT Energy	ZPE	Solvation	System	DFT Energy	ZPE	Solvation
1	−541.37	4.03	−542.04	31	−536.91	4.69	−537.40
2	−544.86	4.36	−545.58	32	−537.58	4.28	−538.01
3	−547.86	4.60	−548.56	33	−538.20	4.69	−538.74
4	−547.77	4.66	−548.43	34	−526.87	4.26	−527.24
5	−533.34	3.89	−533.86	35	−540.75	4.98	−541.27
6	−536.48	4.18	−537.17	36	−525.97	4.24	−526.46
7	−551.57	4.93	−552.18	37	−526.50	4.98	−526.88
8	−536.80	4.19	−537.30	38	−541.06	4.97	−541.66
9	n/a	n/a	n/a	39	−530.91	4.56	−531.31
10	−526.28	3.71	−526.66	40	−529.91	4.57	−530.62
11	−540.84	4.50	−541.50	41	−529.93	4.56	−530.45
12	−540.76	4.52	−541.30	42	−518.87	4.10	−519.22
13	−541.18	4.51	−541.73	43	−534.00	4.86	−534.51
14	−553.86	5.22	−555.04	44	−522.97	4.43	−523.34
15	−540.34	4.52	−540.83	45	−527.18	4.75	−527.59
16	−528.77	4.04	−529.32				
17	−529.03	4.06	−529.57				
18	n/a	n/a	n/a				
19	−543.95	4.79	−544.45				
20	−529.70	4.74	−530.50				
21	−543.76	4.50	−544.62				
22	−533.28	4.34	−533.78				
23	−533.41	4.36	−533.89				
24	−533.11	4.37	−533.61				
25	−547.72	5.07	−548.25				
26	−534.04	4.41	−534.61				
27	−534.74	4.41	−535.20				
28	−532.89	4.38	−533.47				
29	−537.58	4.28	−538.01				
30	−522.81	3.96	−523.19				

S3 Partial charges on the atoms of the intermediates

Table S4: DDEC6 partial charges on the atoms of the intermediates on the pure Cu surface. The last colums is the total charge on the intermediate.

	C 1	C 2	O 1	O 2	O 3	O 4	H 1	H 2	H 3	H 4	Σ
01E	0.46	0.46	−0.41	−0.41	−0.41	−0.41	n/a	n/a	n/a	n/a	−0.73
02E	0.52	0.46	−0.42	−0.41	−0.40	−0.42	0.40	n/a	n/a	n/a	−0.28
03D	0.40	0.40	−0.40	−0.39	−0.40	−0.39	0.36	0.36	n/a	n/a	−0.07
07C	0.14	0.47	−0.43	−0.43	−0.47	−0.39	0.35	0.33	0.37	n/a	−0.05
12A	0.52	−0.24	−0.42	−0.39	−0.43	n/a	0.39	0.34	n/a	n/a	−0.24
18A	−0.16	0.35	−0.40	−0.41	−0.37	n/a	0.11	0.38	0.35	n/a	−0.17
25E	−0.19	0.52	−0.41	−0.41	−0.41	n/a	0.12	0.28	0.38	0.31	0.20
31A	0.11	−0.11	−0.41	−0.36	n/a	n/a	0.07	0.38	0.30	n/a	−0.03
36A	0.13	−0.42	−0.37	n/a	n/a	n/a	0.16	0.34	n/a	n/a	−0.15
40A	0.20	−0.48	−0.35	n/a	n/a	n/a	0.15	0.07	0.37	n/a	−0.05
43A	−0.43	0.24	−0.35	n/a	n/a	n/a	0.17	0.33	0.09	0.17	0.22
44A	−0.20	−0.30	n/a	n/a	n/a	n/a	0.13	0.13	0.11	n/a	−0.12
45A	−0.23	−0.23	n/a	n/a	n/a	n/a	0.14	0.14	0.14	0.14	0.10

Table S5: DDEC6 partial charges on the atoms of the intermediates on the Ni surface with 1 Cu overlayer. The last colums is the total charge on the intermediate.

	C 1	C 2	O 1	O 2	O 3	O 4	H 1	H 2	H 3	H 4	Σ
01E	0.46	0.46	−0.42	−0.42	−0.42	−0.42	n/a	n/a	n/a	n/a	−0.77
02E	0.52	0.46	−0.43	−0.42	−0.41	−0.42	0.40	n/a	n/a	n/a	−0.31
03D	0.43	0.43	−0.43	−0.41	−0.43	−0.41	0.39	0.39	n/a	n/a	−0.04
07C	0.13	0.48	−0.43	−0.44	−0.47	−0.40	0.36	0.34	0.37	n/a	−0.06
12A	0.53	−0.24	−0.41	−0.40	−0.43	n/a	0.39	0.35	n/a	n/a	−0.22
18A	−0.14	0.41	−0.45	−0.44	−0.35	n/a	0.11	0.38	0.36	n/a	−0.12
25E	−0.20	0.52	−0.41	−0.41	−0.41	n/a	0.13	0.28	0.38	0.31	0.19
31A	0.10	−0.11	−0.41	−0.36	n/a	n/a	0.08	0.38	0.30	n/a	−0.03
36A	0.13	−0.42	−0.37	n/a	n/a	n/a	0.17	0.35	n/a	n/a	−0.15
40A	0.20	−0.49	−0.36	n/a	n/a	n/a	0.15	0.07	0.37	n/a	−0.06
42A	−0.26	−0.26	n/a	n/a	n/a	n/a	0.15	0.15	n/a	n/a	−0.22
44A	−0.20	−0.30	n/a	n/a	n/a	n/a	0.13	0.14	0.11	n/a	−0.12
45A	−0.24	−0.24	n/a	n/a	n/a	n/a	0.14	0.14	0.14	0.14	0.09

Table S6: DDEC6 partial charges on the atoms of the intermediates on the Ni surface with 2 Cu overlayers. The last colums is the total charge on the intermediate.

	C 1	C 2	O 1	O 2	O 3	O 4	H 1	H 2	H 3	H4	Σ
01E	0.46	0.46	−0.41	−0.41	−0.41	−0.41	n/a	n/a	n/a	n/a	−0.73
02E	0.52	0.46	−0.42	−0.41	−0.40	−0.42	0.40	n/a	n/a	n/a	−0.28
03F	0.36	0.36	−0.40	−0.39	−0.40	−0.39	0.37	0.37	n/a	n/a	−0.14
07C	0.13	0.48	−0.43	−0.43	−0.47	−0.39	0.33	0.37	0.36	n/a	−0.04
12A	0.53	−0.24	−0.42	−0.39	−0.43	n/a	0.39	0.35	n/a	n/a	−0.23
18D	−0.26	0.46	−0.40	−0.49	−0.28	n/a	0.10	0.37	0.36	n/a	−0.15
25E	−0.20	0.52	−0.41	−0.41	−0.41	n/a	0.13	0.28	0.38	0.314	0.21
32A	0.61	−0.69	−0.44	−0.41	n/a	n/a	0.14	0.29	0.39	n/a	−0.12
38A	0.61	−0.62	−0.42	−0.41	n/a	n/a	0.19	0.35	0.34	0.188	0.24
41B	−0.43	0.12	−0.38	n/a	n/a	n/a	0.16	0.14	0.35	n/a	−0.04
43A	−0.42	0.24	−0.35	n/a	n/a	n/a	0.17	0.32	0.09	0.175	0.23
44A	−0.21	−0.29	n/a	n/a	n/a	n/a	0.13	0.13	0.11	n/a	−0.13
45A	−0.23	−0.23	n/a	n/a	n/a	n/a	0.14	0.14	0.14	0.142	0.10

S4 Sum of Bond Orders

Table S7: Cu surface

	C 1	C 2	O 1	O 2	O 3	O 4	H 1	H 2	H 3	H 4	C-C BO
01E	4.12	4.12	2.35	2.35	2.34	2.34	n/a	n/a	n/a	n/a	0.78
02E	4.03	4.21	2.36	2.34	2.34	2.22	0.82	n/a	n/a	n/a	0.83
03D	4.12	4.12	2.32	2.37	2.32	2.37	0.88	0.88	n/a	n/a	1.02
07C	4.00	4.13	2.25	2.35	2.29	2.38	0.90	0.96	0.88	n/a	1.02
12A	4.08	4.04	2.35	2.48	2.19	n/a	0.88	0.94	n/a	n/a	1.24
18B	4.14	4.24	2.57	2.50	2.31	n/a	1.01	0.85	0.90	n/a	1.60
25E	4.07	4.07	2.24	2.32	2.35	n/a	1.00	1.05	0.88	0.99	1.24
31A	3.99	4.13	2.37	2.26	n/a	n/a	1.04	0.86	1.02	n/a	1.46
36A	3.94	3.88	2.29	n/a	n/a	n/a	0.98	0.90	n/a	n/a	1.47
40A	3.99	4.08	2.52	n/a	n/a	n/a	1.01	0.98	0.86	n/a	1.72
43A	3.93	3.97	2.39	n/a	n/a	n/a	1.01	0.93	0.97	0.99	1.53
44A	3.86	3.96	n/a	n/a	n/a	n/a	1.01	0.98	1.06	n/a	1.69
45A	3.89	3.89	n/a	n/a	n/a	n/a	1.00	1.00	1.00	1.00	1.51

Table S8: Ni surface with 1 Cu overlayer

	C 1	C 2	O 1	O 2	O 3	O 4	H 1	H 2	H 3	H 4	C-C BO
01E	4.12	4.12	2.34	2.34	2.34	2.34	n/a	n/a	n/a	n/a	0.77
02E	4.03	4.22	2.36	2.34	2.34	2.22	0.82	n/a	n/a	n/a	0.83
03D	4.11	4.11	2.23	2.53	2.23	2.52	0.84	0.84	n/a	n/a	0.99
07C	4.00	4.13	2.25	2.35	2.29	2.37	0.89	0.95	0.88	n/a	1.01
12A	4.08	4.01	2.36	2.47	2.18	n/a	0.88	0.92	n/a	n/a	1.22
18A	4.03	4.28	2.45	2.55	2.30	n/a	1.01	0.85	0.88	n/a	1.47
25E	4.09	4.07	2.23	2.31	2.34	n/a	0.99	1.04	0.87	0.99	1.24
31A	3.99	4.15	2.37	2.26	n/a	n/a	1.04	0.86	1.02	n/a	1.47
36A	3.95	3.90	2.28	n/a	n/a	n/a	0.97	0.90	n/a	n/a	1.48
40A	4.00	4.09	2.52	n/a	n/a	n/a	1.01	0.98	0.86	n/a	1.73
42A	3.94	3.94	n/a	n/a	n/a	n/a	0.97	0.97	n/a	n/a	1.66
44A	3.86	3.97	n/a	n/a	n/a	n/a	1.01	0.98	1.06	n/a	1.70
45A	3.89	3.89	n/a	n/a	n/a	n/a	1.00	1.00	1.00	1.00	1.52

Table S9: Ni surface with 2 Cu overlayers

	C 1	C 2	O 1	O 2	O 3	O 4	H 1	H 2	H 3	H 4	C-C BO
01E	4.12	4.12	2.34	2.34	2.34	2.34	n/a	n/a	n/a	n/a	0.77
02E	4.03	4.22	2.35	2.34	2.35	2.22	0.82	n/a	n/a	n/a	0.83
03F	4.13	4.13	2.35	2.35	2.35	2.35	0.87	0.87	n/a	n/a	1.11
07C	3.99	4.13	2.24	2.35	2.29	2.37	0.89	0.95	0.88	n/a	1.01
12A	4.07	4.02	2.35	2.46	2.19	n/a	0.88	0.92	n/a	n/a	1.23
18D	3.98	4.28	2.58	2.21	2.63	n/a	1.05	0.88	0.86	n/a	1.56
25E	4.08	4.06	2.23	2.31	2.33	n/a	0.99	1.04	0.88	0.98	1.23
32A	4.10	4.10	2.18	2.28	n/a	n/a	1.07	1.03	0.85	n/a	1.49
38A	4.06	3.99	2.29	2.30	n/a	n/a	0.99	0.91	0.93	1.00	1.41
41B	3.96	3.94	2.41	n/a	n/a	n/a	1.03	1.01	0.87	n/a	1.52
43A	3.93	3.97	2.38	n/a	n/a	n/a	1.00	0.94	0.97	0.99	1.52
44A	3.88	3.94	n/a	n/a	n/a	n/a	1.01	0.99	1.06	n/a	1.67
45A	3.89	3.89	n/a	n/a	n/a	n/a	1.00	1.00	1.00	1.00	1.51
